# Derivation of a pragmatic three-view protocol for point-of-care transesophageal echocardiography in the cardiovascular ICU: a real-world cohort study

**DOI:** 10.1186/s13054-026-05925-x

**Published:** 2026-03-01

**Authors:** Kimito Minami, Takashi Saga, Mitsuharu Kato, Daisuke Nakayama, Masahiro Morinaga, Tatsutoshi Shimatani, Muneyuki Takeuchi

**Affiliations:** 1 Department of Critical Care Medicine, National Cerebral and Cardiovascular Center, 6-1 Kishibeshinmachi, Suita, 564-8565 Osaka Japan; 2 Department of Anesthesiology, National Cerebral and Cardiovascular Center, 6-1 Kishibeshinmachi, Suita, 564-8565 Osaka Japan

**Keywords:** Point-of-care transesophageal echocardiography, Intensive care unit, Postoperative cardiac surgery, Hemodynamic instability, Mechanical circulatory support

## Abstract

**Background:**

Rapid identification of the cause of hemodynamic instability is essential in the cardiovascular intensive care unit (CV-ICU), and transesophageal echocardiography provides superior diagnostic image quality compared with transthoracic echocardiography in this setting. However, point-of-care transesophageal echocardiography (POCUS-TEE) is not routinely used, partly because of perceived training barriers. We therefore sought to summarize the etiologies of hemodynamic instability in the CV-ICU and to identify a pragmatic minimal set of TEE views required for their diagnosis.

**Methods:**

This single-center retrospective study evaluated consecutive adult cardiovascular ICU admissions over five years. All POCUS-TEE examinations were performed as part of routine clinical care at the discretion of the treating team. Examinations in patients with hemodynamic instability were analyzed to identify the “first informative view” supporting the diagnosis. A cumulative coverage analysis was performed to derive a minimal-view set. Inter-rater reproducibility was assessed for view acquisition and management decisions.

**Results:**

Among 6,898 admissions, POCUS-TEE was performed in 353 examinations (5.1%), predominantly in patients with higher severity scores compared to those without TEE. In preoperative stable patients (*n* = 37), POCUS-TEE altered planned surgery in 6 patients (16.2%). In postoperative stable patients (*n* = 101), management changes included optimization of mechanical support and anticoagulation. In postoperative-unstable examinations (*n* = 238), 79.4% prompted procedural interventions. A derived minimal three-view set (mid-esophageal bicaval, mid-esophageal four-chamber, and transgastric mid-short-axis) achieved 92.4% diagnostic coverage in unstable examinations. While the specific view acting as the first diagnostic window varied due to acquisition sequence (κ 0.06–0.11), the core three-view set was highly reproducible across raters (Jaccard similarity coefficient 0.60), and agreement on management decisions was moderate. No procedure-related complications were observed during bedside POCUS-TEE.

**Conclusions:**

POCUS-TEE frequently drives urgent interventions in postoperative hemodynamic instability. Although the sequence of diagnostic capture is operator-dependent, a simple three-view protocol provides > 90% diagnostic coverage. This derivation supports a physiology-based, limited-view approach for training, early competency development, and resuscitation in cardiovascular critical care.

**Supplementary Information:**

The online version contains supplementary material available at 10.1186/s13054-026-05925-x.

## Introduction

Point-of-care ultrasound (POCUS) has become an essential diagnostic modality in the intensive care unit (ICU), enabling real-time, bedside assessment of cardiovascular, pulmonary, and abdominal pathology [1]. Among various POCUS applications, transesophageal echocardiography (TEE) provides superior image quality and anatomical detail. Particularly in the postoperative CV-ICU setting, TTE windows are frequently compromised by surgical dressings, chest tubes, subcutaneous emphysema, and mechanical ventilation, rendering adequate visualization frequently suboptimal or non-diagnostic [2]. Consequently, POCUS-TEE is deployed when TTE is non-diagnostic or implies diagnostic uncertainty. In the cardiovascular postoperative ICU (CV-ICU), where patients often present with complex hemodynamic disturbances, valvular interventions, and mechanical circulatory support (MCS) devices, TEE has the potential to serve as a critical adjunct to clinical decision-making [3–5]. Despite these advantages, the routine integration of TEE as a POCUS tool in the ICU remains limited. Barriers include the perception of invasiveness, lack of operator training, institutional barriers, and absence of simplified protocols comparable to other bedside ultrasound applications, such as the Focused Assessment with Sonography for Trauma (FAST) [6–14]. While prior studies have described TEE’s utility in isolated settings such as cardiac arrest or shock evaluation [3, 15], few studies have evaluated its use across consecutive ICU patients. Moreover, limited contemporary cohort descriptions focus specifically on consecutive CV-ICU patients cared for in the era of durable left ventricular assist devices (LVADs) and extracorporeal support.

Accordingly, our primary objective was to characterize the indications, diagnostic yield, and management implications of bedside POCUS-TEE across consecutive CV-ICU patients and, informed by these observations, to develop a pragmatic imaging set that captures the majority of clinically relevant diagnostic information needed to clarify the etiology of hemodynamic instability in this setting.

## Materials and methods

### Study design and ethical considerations

We conducted a single-center, observational cohort study of consecutive adult patients undergoing POCUS-TEE in a CV-ICU between July 2019 and May 2025. The protocol adhered to the STROBE reporting guideline. All point-of-care transesophageal echocardiography examinations were performed as part of routine clinical care at the discretion of the treating team and were not mandated by the study protocol. The present analysis was a retrospective review of these clinically indicated examinations. The institutional ethics committee approved the study on 11 April 2025 (approval number: R25002) and waived the requirement for informed consent, as the study involved the retrospective analysis of de-identified data with an opt-out mechanism publicly posted.

### Participants and analytic unit

Eligible cases were adults (≥ 18 years) admitted to the CV-ICU who received a bedside POCUS-TEE for diagnostic clarification or procedure guidance. Operating-room–only examinations were excluded. The analytic unit was the individual examination (an individual patient could contribute > 1 examination). For descriptive subgroup summaries, examinations were stratified by (1) perioperative timing (preoperative vs. postoperative) and (2) hemodynamic status at the start of TEE (stable vs. unstable). Unstable was defined by ≥ 1 of the following criteria: (i) documented shock or persistent hypotension requiring active resuscitation; (ii) ongoing vasopressor and/or inotrope infusion intensification; (iii) extracorporeal cardiopulmonary resuscitation (ECPR)/cardiopulmonary resuscitation (CPR) in progress or extracorporeal membrane oxygenation (ECMO) cannulation/urgent cannula manipulation underway; (iv) the treating team designating the patient as hemodynamically unstable in the contemporaneous record. When none of the above criteria were present, the patient was categorized as stable.

### Indications, image acquisition, and data sources

At the time of scanning, operators selected one or more predefined clinical indications (e.g., undifferentiated hypotension/low cardiac output, pericardial effusion/tamponade, ventricular dysfunction/ischemia, valve assessment, intracardiac/aortic thrombus or aortic pathology, mechanical circulatory support [MCS] evaluation, cannulation guidance including ECPR/VA-ECMO). Examinations were performed with an adult multiplane TEE probe at the bedside using a focused technique. Standard two-dimensional imaging was supplemented by color and spectral Doppler at the operator’s discretion. Clinical variables (demographics, surgical context, vasoactive/MCS status) and outcomes were abstracted from the electronic health record. Indications, echocardiographic findings, and first informative views were obtained from structured TEE reports and archived images.

### Definition of the first informative view

We defined the first informative view as the tomographic window that first provided sufficient echocardiographic evidence supporting the ultimate working diagnosis during the examination. The term is descriptive and does not imply that this single view alone was sufficient for management decisions; final diagnoses and actions reflected the full clinical and imaging synthesis. Operators recorded the first informative view prospectively at the time of scanning using a standardized list mapped to the ASE/SCA comprehensive 28-view taxonomy (e.g., mid-esophageal [ME] four-chamber [ME 4 C], ME bicaval, transgastric [TG] mid-short-axis [mid-SAX], upper-esophageal [UE] aortic arch views) [16, 17]. If two views were considered equally informative at essentially the same time, the earlier standardized window in the acquisition sequence was recorded.

### Outcomes

(1) Pre-specified descriptive endpoints included:

1. the distribution of diagnostic categories identified by POCUS-TEE across strata;
2. the distribution of first informative views overall and by indication; and.
3. management actions temporally linked to POCUS-TEE, grouped as surgical/procedural, non-surgical, or no change based on contemporaneous chart documentation.

Identification of first informative views and management actions was based on contemporaneous clinical records, including procedure notes and progress notes, and attribution to POCUS-TEE was performed descriptively and retrospectively.

To further assess the IRR of the first informative view designation and its associated clinical interpretation, 100 postoperative-unstable examinations were randomly sampled from the full cohort. Three clinician-sonographers (raters 1–3), blinded to each other’s evaluation and to clinical outcomes, independently reviewed the archived image sets and (1) selected the first informative view and (2) classified the temporally linked management action into one of three categories (surgical/procedural, non-surgical adjustment, or no intervention). Agreement between each rater and the reference classification from the full cohort was quantified using the proportion of concordant ratings for both the first informative view and management action categories. Agreement with the reference classification was assessed using raw concordance and Cohen’s κ, interpreted using standard benchmarks (slight: <0.20; fair: 0.21–0.40; moderate: 0.41–0.60). Variation across raters was summarized descriptively.

(2) Limited-view coverage analysis and reproducibility:

In the postoperative-unstable cohort, we quantified how many tomographic windows are needed to cover most examinations in which the first informative view belonged to a candidate view set. For each ASE/SCA standard view, we counted how often it was recorded as the first informative view and rank-ordered views by this frequency. Cumulative coverage was then calculated as views were added sequentially in this order. The reference minimal-view set was defined as the smallest number of views required to reach a cumulative coverage of ≥ 90% of postoperative-unstable examinations. Coverage was defined as the proportion of examinations whose first informative view belonged to the candidate set and does not represent a diagnostic performance metric. To assess the reproducibility of this minimal-view set across observers, we used the subset of postoperative-unstable examinations that underwent independent blinded re-reading by three clinician–sonographers as part of the inter-rater reliability assessment. For each rater, we repeated the stepwise frequency ranking and cumulative coverage calculation using that rater’s classification of first informative views and defined a rater-specific minimal-view set as the smallest number of views achieving ≥ 90% coverage in the re-read sample. We summarized the overlap between rater-specific and reference sets as the number and percentage of views in common. To further quantify the similarity between the view sets obtained by each rater and the reference minimal set, we calculated the Jaccard similarity coefficient, defined as the size of the intersection divided by the size of the union of view sets (Jaccard = *|A ∩ B| / |A ∪ B|*), ranging from 0 to 1, with higher values indicating greater overlap.

### Safety endpoint

TEE-related complications were defined as adverse events temporally associated with transesophageal echocardiography probe insertion or manipulation. Identification of complications was based on electronic medical record review, including structured chart review of procedure notes, nursing records, and post-procedure clinical documentation. The assessment window extended from the start of probe insertion until 24 h after the examination. Events occurring outside this window were considered unrelated unless explicitly attributed to TEE in the medical record.

### Statistical analysis

Continuous variables are presented as median (IQR) and categorical variables as counts (%). Between-group summaries are descriptive; no hypothesis testing or multivariable modeling was planned. Cohen’s κ was calculated using R version 4.3.1 (R Foundation for Statistical Computing, Vienna, Austria). Analyses included all eligible consecutive examinations during the study period; missingness is reported per variable, and no imputation was performed. No formal sample-size calculation was performed; the cohort includes all eligible consecutive examinations over 5 years, providing a pragmatic precision-based sample to estimate diagnostic yields and complication rates and to explore view-to-diagnosis relationships.

## Results

### Study population and selection characteristics

Among 6,898 CV-ICU admissions, 353 patients (5.1%) underwent at least one bedside POCUS-TEE (preoperative-stable *n* = 37; postoperative-stable *n* = 101; postoperative-unstable *n* = 215 with 238 examinations). Baseline characteristics by POCUS-TEE exposure are summarized in Table 1. Patients selected for POCUS-TEE exhibited higher disease acuity compared to those who did not, as indicated by higher APACHE II scores (median 22 vs. 19) and SOFA scores (median 11 vs. 8). The TEE cohort also had a higher prevalence of mechanical circulatory support (37.7% vs. 5.9%), reflecting a population with complex hemodynamic needs where standard monitoring was likely insufficient.

**Table 1:** Baseline characteristics by exposure to POCUS-TEE during the index CV-ICU stay

	Underwent POCUS-TEE (*N* = 353)	No POCUS-TEE (*N* = 6545)	Missing (%)
Male sex	218 (61.8%)	4121 (63.0%)	0
Age, years	67 [53, 78]	72 [60, 79]	0
Height, cm	163 [157, 171]	163 [155, 170]	2.8
Weight, kg	61 [51, 72]	60 [51, 69]	1.8
Hypertension	279 (79.0%)	4509 (68.9%)	0
Dyslipidemia	101 (28.6%)	1912 (29.2%)	0
Diabetes	228 (64.6%)	4444 (67.9%)	0
HbA1c, %	6.0 [5.6, 6.5]	5.9 [5.6, 6.4]	7.8
Creatinine, mg/dL	1.05 [0.82, 1.47]	0.93 [0.75, 1.17]	0.1
SOFA score	11 [9, 12]	8 [5, 10]	4.8
APACHE II score	22 [16, 27]	19 [13, 24]	29.9
Index surgical procedure			0
MCS	133 (37.7%)	282 (5.9%)	
Valve	77 (21.8%)	2236 (34.2%)	
Coronary	45 (12.7%)	935 (14.3%)	
Vascular	39 (11.0%)	931 (14.2%)	
HTx	27 (7.6%)	140 (2.1%)	
Intervention	9 (2.5%)	939 (14.3%)	
Others	23 (6.5%)	981 (15.0%)	

Continuous variables are reported as median [IQR]; categorical variables are n (%). POCUS-TEE, point-of-care transesophageal echocardiography; SOFA, Sequential Organ Failure Assessment; APACHE II, Acute Physiology and Chronic Health Evaluation II; MCS, mechanical circulatory support; HTx, heart transplantation

### Safety

There were no major procedure-related complications (e.g., esophageal perforation, significant oropharyngeal bleeding, or hemodynamic collapse directly attributable to probe insertion) recorded in the 353 examinations.

### Indications, diagnoses, and management actions

In the preoperative-stable group (*n* = 37), indications were predominantly valve evaluation (*n* = 34) and thrombus evaluation (*n* = 3). POCUS-TEE prompted changes in surgical planning in 6/37 (16.2%) overall: omission of planned valve surgery in 3/34 (8.8%) valve-evaluation cases, and addition or omission of thrombectomy in all 3/3 (100%) thrombus-evaluation cases (two added, one omitted). No emergent hemodynamic interventions were recorded in this stratum (Supplemental Table 1).

In the postoperative-stable group, common indications included MCS evaluation, thrombus evaluation, ischemia evaluation, and pericardial effusion evaluation, with both non-surgical adjustments (e.g., device settings, vasoactive/ventilator titration) and selected surgical interventions when warranted (Supplemental Table 2).

In the postoperative-unstable group, indications were dominated by low cardiac output syndrome (LCOS) and ECPR/VA-ECMO cannulation guidance (Table 2). In this setting, POCUS-TEE was frequently associated with downstream action: for LCOS, 148/209 (70.8%) underwent surgical/procedural intervention, including re-sternotomy for cardiac tamponade/pericardial effusion when identified (103/107, 96.3%), while non-surgical adjustments (e.g., vasoactive therapy, device optimization) were also common.

**Table 2:** Postoperative-unstable cohort: management impact of POCUS-TEE by indication with nested TEE-derived diagnoses

Indication	Diagnosis	Total number	No change, *n* (%)	Non-surgical change, *n* (%)	Surgical/procedural, *n* (%)	Change types
LCOS		209	13 (6.2%)	48 (23.0%)	148 (70.8%)	
	— Cardiac tamponade/pericardial effusion	107	0 (0.0%)	4 (3.7%)	103 (96.3%)	Re-sternotomy/ re-exploration (*n* = 103); ECPR (*n* = 6); Vasopressor therapy (*n* = 4)
	— Hypovolemia	21	0 (0.0%)	21 (100.0%)	0 (0.0%)	Fluid bolus (*n* = 21)
	— Drainage cannula malfunction	20	0 (0.0%)	0 (0.0%)	20 (100.0%)	ECMO/device cannula repositioning (*n* = 20)
	— Left ventricular failure	15	2 (13.3%)	9 (60.0%)	4 (26.7%)	Inotropic therapy (*n* = 13); IABP insertion (*n* = 4)
	— Right ventricular failure	14	0 (0.0%)	9 (64.3%)	5 (35.7%)	Inotropic therapy (*n* = 9); ECPR (*n* = 4); ECMO/device cannula repositioning (*n* = 1)
	— Acute myocardial infarction	10	0 (0.0%)	0 (0.0%)	10 (100.0%)	CABG (*n* = 7); ECPR (*n* = 4); PCI (*n* = 1)
	— Impella malposition	3	0 (0.0%)	0 (0.0%)	3 (100.0%)	Impella repositioning (*n* = 3)
	— Vasoplegia/distributive shock	2	0 (0.0%)	2 (100.0%)	0 (0.0%)	Vasopressor therapy (*n* = 2)
	— Hemothorax	2	0 (0.0%)	0 (0.0%)	2 (100.0%)	Drainage/ chest tube (*n* = 2)
	— Left ventricular outflow tract stenosis	2	0 (0.0%)	2 (100.0%)	0 (0.0%)	Vasopressor therapy (*n* = 2)
	— Abdominal bleeding	1	0 (0.0%)	0 (0.0%)	1 (100.0%)	Laparotomy (*n* = 1)
	— Abdominal compartment syndrome	1	0 (0.0%)	1 (100.0%)	0 (0.0%)	Neuromuscular blocking agent bolus (*n* = 1)
	— Pulmonary valve regurgitation	1	1 (100.0%)	0 (0.0%)	0 (0.0%)	
	— No TEE-identified etiology of LCOS	10	10 (100.0%)	0 (0.0%)	0 (0.0%)	
TEE-guided V-A ECMO placement		26	0 (0.0%)	0 (0.0%)	26 (100.0%)	
	— TEE-guided V-A ECMO placement	26	0 (0.0%)	0 (0.0%)	26 (100.0%)	ECPR (*n* = 26)
TEE-guided IABP insertion		2	0 (0.0%)	0 (0.0%)	2 (100.0%)	
	— TEE-guided IABP insertion	2	0 (0.0%)	0 (0.0%)	2 (100.0%)	IABP insertion (*n* = 2)
TEE-guided V-V ECMO placement		1	0 (0.0%)	0 (0.0%)	1 (100.0%)	
	— TEE-guided V-V ECMO placement	1	0 (0.0%)	0 (0.0%)	1 (100.0%)	V-V ECMO initiation (*n* = 1)

Rows are grouped by clinical indications (top level) with corresponding diagnoses nested underneath. Values are n (%) for No change, Non-surgical change, and Surgical/Procedural intervention after POCUS-TEE; percentages use the row total as the denominator. The rightmost column lists the management change categories observed within each row (ties allowed). POCUS-TEE, point-of-care transesophageal echocardiography; MCS, mechanical circulatory support; IABP, intra-aortic balloon pump; ECMO, extracorporeal membrane oxygenation; PCI, percutaneous coronary intervention

### First informative view summaries

**Fig. 1:**
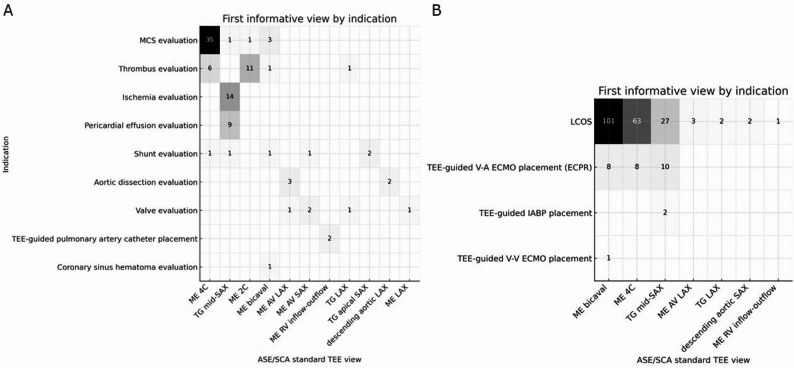
First informative view by indication in the postoperative-stable cohort and postoperative-unstable cohort. **A**, postoperative-stable cohort; **B**, postoperative-unstable cohort. Heatmaps showing each clinical indication (rows), the TEE view that yielded the first informative finding (columns). Numbers within cells are counts of examinations in which that view provided the first informative finding for the stated indication; darker shading denotes higher counts, and empty/zero cells indicate no informative findings for that combination. Abbreviations: ME, mid-esophageal; TG, transgastric; UE, upper-esophageal; 4 C/2 C, four-/two-chamber; LAX/SAX, long-/short-axis; AV, aortic valve; RV, right ventricle; MCS, mechanical circulatory support; LCOS, low cardiac output syndrome; ECPR, extracorporeal cardiopulmonary resuscitation; IABP, intra-aortic balloon pumping; V-A/V-V ECMO, veno-arterial/veno-venous extracorporeal membrane oxygenation

Across postoperative examinations, mid-esophageal cardiac windows were most often recorded as the first informative view. In postoperative-unstable exams, ME bicaval and ME 4 C predominated for obstructive and right-sided etiologies (e.g., tamponade, RV failure), while transgastric (TG) mid-SAX frequently provided the first informative evidence of ventricular dysfunction/ischemia. In postoperative-stable assessments, incremental contributions came from left-heart structural views (ME 2 C and ME AV LAX/SAX) consistent with broader, question-driven evaluations of valvular and chamber abnormalities. Heatmaps by indication are shown in Fig. 1.

### Limited-view coverage analysis

Rank-ordering views by frequency of serving as the first informative view, core three views (ME bicaval, ME 4 C, and TG mid-SAX) achieved ≥ 90% cumulative coverage of postoperative-unstable examinations, together accounting for 92.4% of examinations and forming the 90% minimal-view set.

### Rater-specific reproducibility

In 100 randomly sampled postoperative-unstable examinations re-read independently by three clinician-sonographers, each rater-specific minimal-view set met the predefined ≥ 90% coverage threshold (Supplemental Fig. 1):

- Rater 1: core three + ME AV LAX + ME RV inflow-outflow (92.0% coverage).
- Rater 2: core three + ME AV LAX + ME AV SAX (93.0% coverage).
- Rater 3: core three + ME AV LAX + ME AV SAX (90.0% coverage).

All raters consistently included the same core three views. Similarity with the reference core set was high (Jaccard similarity coefficient = 0.60 for all raters), indicating operator-independent reproducibility of the pragmatic minimal-view sequence.

### Inter-rater reliability analyses

Agreement with the clinical reference for identifying the first informative view showed a raw agreement of 0.28–0.31 across raters, with κ values ranging from 0.06 to 0.11. For management decision classification, raw agreement ranged from 0.55 to 0.56 across raters. κ estimates for management decisions were unstable due to substantial category imbalance, with relatively few no-intervention classifications; interpretation therefore relied primarily on raw agreement.

## Discussion

### Summary of findings

In this contemporary CV-ICU cohort, bedside POCUS-TEE was frequently deployed for rapid hemodynamic clarification, particularly in postoperative instability, and was associated with actionable management in most unstable cases. Descriptively, a small number of standard mid-esophageal and transgastric windows accounted for most first informative findings. Specifically, ME bicaval, ME 4 C, and TG mid-SAX collectively explained > 90% of diagnostic clarifications in unstable patients in our unit, while left-heart structural views (ME 2 C, ME AV LAX/SAX) provided incremental value in stable or question-driven examinations. These findings reinforce a physiologic rationale in post-cardiac surgery circulatory failure, consistent with prior work demonstrating the high yield of targeted critical-care TEE in shock states where transthoracic windows are limited [4, 5, 15]. 

### Interpretation and implications for clinical workflow

These results support integrating POCUS-TEE into resuscitation workflows in high-acuity environments. ME bicaval and ME 4 C rapidly assess obstructive and right-sided causes of shock, including pericardial tamponade and RV failure, while TG mid-SAX enables efficient assessment of biventricular function, septal shift, and ischemia in LCOS. In contrast, stable postoperative patients benefit from broader, question-driven protocols that incorporate ME 2 C and targeted aortic valve imaging to evaluate prosthetic dysfunction, shunts, or thrombus. Our findings thereby refine procedural prioritization: a context-specific, three-view starting sequence during initial resuscitation, with rapid expansion as diagnostic questions evolve. Accordingly, the proposed three-view protocol is intended to support initial physiologic triage and does not replace a comprehensive or question-driven echocardiographic examination as clinical evaluation progresses.

### Reproducibility: view sequence versus view set

We observed low inter-rater agreement (κ < 0.20) regarding which specific view was designated as the “first informative view”. This finding is expected and likely reflects the stochastic nature of the scanning sequence rather than a lack of utility. In an emergent setting, the “first” diagnostic view depends on which window the operator chooses to acquire first. For example, a pericardial tamponade might be identified first on a transgastric view by one operator, but on a mid-esophageal view by another, yet both reach the same diagnosis and management decision. Crucially, while the sequence varied, the content of the minimal-view set did not. All blinded raters independently derived view sets that included the same core three views (ME bicaval, ME 4 C, TG mid-SAX), and these sets consistently achieved high coverage of actionable pathology. Moreover, high Jaccard similarity (0.60) suggests that the proposed protocol is robust and operator-independent, even though the exact timing of diagnostic recognition varies. The moderate agreement on management decisions further supports that POCUS-TEE leads to consistent clinical actions.

### Comparison with and extension of prior work

Earlier guidelines and evaluations emphasized complete diagnostic protocols requiring extensive training volumes for competency [10, 17–22]. Our data extends this field by demonstrating how a limited-view, physiology-first approach achieves high diagnostic utility specifically in postoperative hemodynamic compromise, an ICU population less represented in earlier general critical-care TEE studies. Where previous studies relied primarily on expert consensus view sets, our stepwise approach empirically identifies a minimal-view core tailored to the shock etiologies most prevalent in surgical ICUs. This approach complements rather than supersedes established comprehensive protocols and aligns with emerging training proposals advocating pragmatic, tiered skill acquisition [18, 22, 23]. These results carry key implications for training and dissemination. The early mastery of three core views—ME bicaval, ME 4 C, TG mid-SAX—could address the majority of instability in many CV-ICU environments, lowering the entry barrier for intensivists seeking competency [7–9]. As learners progress to stable, question-driven examinations, structured inclusion of ME 2 C and aortic-valve LAX/SAX windows can expand diagnostic breadth. This staged, needs-based curriculum is consistent with competency frameworks proposed by the American Society of Echocardiography and critical-care societies, supporting a shift from niche expertise toward broader adoption.

### Limitations

Several limitations warrant consideration. First, this was a single-center retrospective study conducted in a tertiary cardiovascular ICU, and case inclusion depended on the treating teams’ decision to request POCUS-TEE. Accordingly, the findings should be interpreted within this CV-ICU context and may not be directly generalizable to medical or mixed ICU populations with different shock phenotypes, and causal inference cannot be established. Second, the attribution of management changes was abstracted from contemporaneous charting rather than prospectively adjudicated, raising the possibility of misclassification and attribution bias. Third, several indication strata contained relatively few cases, limiting precision and the power for subgroup analyses. Fourth, our stepwise coverage analysis is a context-specific, descriptive approach: first informative views are conditional on the scanning sequence, operator judgment, and documentation practices, and the resulting coverage figures do not establish diagnostic sufficiency or generalizable performance. In addition, not all examinations had a recorded first informative view, so coverage estimates were restricted to those with documentation and may be sensitive to documentation patterns. Fifth, while the derived three-view protocol covers the majority of hemodynamic etiologies, a limited-view approach inherently risks missing focal pathologies outside these planes (e.g., apical thrombus not visualized in TG mid-SAX, or aortic atheroma). Therefore, this protocol is intended for rapid hemodynamic triage, not as a substitute for comprehensive structural evaluation. Sixth, examinations were performed by clinicians with specific training and established experience in critical care TEE. The diagnostic yield and time-to-acquisition of these core views may differ for novice operators or in institutions with different training thresholds, limiting generalizability to less experienced centers. Finally, management changes and clinical working diagnoses were abstracted retrospectively from contemporaneous clinical documentation and were not systematically validated against an independent gold standard (e.g., comprehensive TEE, computed tomography, or autopsy) in all cases, raising the possibility of misclassification, attribution bias, and verification bias. Addressing these limitations will require prospective validation of this triage-oriented protocol in similar CV-ICU populations, particularly with respect to feasibility and workflow integration.

## Conclusions

Bedside POCUS-TEE frequently informed time-sensitive management in postoperative hemodynamic instability. A simple, three-view core (ME bicaval, ME 4 C, TG mid-SAX) captured > 90% of first informative findings in this population and remained reproducible across readers, supporting its use as a pragmatic starting sequence for resuscitation and as an educational scaffold for developing TEE competency. Diagnostic sufficiency is not implied, and comprehensive imaging remains essential as diagnostic questions evolve.

## Electronic Supplementary Material

Below is the link to the electronic supplementary material.

Supplementary Material 1

## Data Availability

The data supporting the findings of this study are available from the corresponding author, KM, upon reasonable request. KM had full access to all the data in the study and takes responsibility for the integrity of the data and the accuracy of the data analysis.

## Abbreviations

2C: Two-chamber
4C: Four-chamber
APACHE II: Acute Physiology and Chronic Health Evaluation II
ASE/SCA: American Society of Echocardiography/Society of Cardiovascular Anesthesiologists
AV: Aortic valve
CABG: Coronary artery bypass grafting
CPR: Cardiopulmonary resuscitation
CV-ICU: Cardiovascular intensive care unit
ECPR: Extracorporeal cardiopulmonary resuscitation
ECMO: Extracorporeal membrane oxygenation
FAST: Focused Assessment with Sonography for Trauma
HTx: Heart transplantation
IABP: Intra-aortic balloon pumping
ICU: Intensive care unit
IQR: Interquartile range
LAX: Long-axis
LCOS: Low cardiac output syndrome
LV: Left ventricle / left ventricular
LVAD: Left ventricular assist device
LVOT: Left ventricular outflow tract
ME: Mid-esophageal
MCS: Mechanical circulatory support
OR: Operating room
PA catheter: Pulmonary artery catheter
PCI: Percutaneous coronary intervention
POCUS-TEE: Point-of-care transesophageal echocardiography
RV: Right ventricle / right ventricular
RVAD: Right ventricular assist device
SAX: Short-axis
SOFA: Sequential Organ Failure Assessment
STROBE: Strengthening the Reporting of Observational Studies in Epidemiology
TG: Transgastric
UE: Upper-esophageal
VSP: Ventricular septal perforation
