# Supplementary material for: Derivation of a pragmatic three-view protocol for point-of-care transesophageal echocardiography in the cardiovascular ICU: a real-world cohort study
Source: Crit Care. 2026 Mar 1;30:154. doi: 10.1186/s13054-026-05925-x (PMC13059169; doi:10.1186/s13054-026-05925-x)
Supplement: Supplementary file 1 — Supplementary Material 1 [file 13054_2026_5925_MOESM1_ESM.docx]

| **Indication** | **Diagnosis** | **Total number** | **Surgical plan change, n (%)** | **Change details** |
| --- | --- | --- | --- | --- |
| Valve evaluation |  | 34 | 3 (8.8%) |  |
|  | — Definite infective endocarditis (aortic valve) | 6 | 0 (0.0%) |  |
|  | — Definite infective endocarditis (aortic and mitral valve) | 3 | 0 (0.0%) |  |
|  | — Definite infective endocarditis (mitral valve) | 3 | 0 (0.0%) |  |
|  | — Definite infective endocarditis (prosthetic mitral valve) | 2 | 0 (0.0%) |  |
|  | — Definite infective endocarditis (RV pacemaker lead) | 2 | 0 (0.0%) |  |
|  | — Definite infective endocarditis (prosthetic aortic valve) | 1 | 0 (0.0%) |  |
|  | — Rejected infective endocarditis | 3 | 3 (100.0%) | Planned valve surgery omitted (n = 3) |
|  | — Degenerative valvular disease (mitral regurgitation) | 6 | 0 (0.0%) |  |
|  | — Degenerative valvular disease (aortic prosthetic valve failure) | 2 | 0 (0.0%) |  |
|  | — Degenerative valvular disease (aortic stenosis) | 2 | 0 (0.0%) |  |
|  | — Degenerative valvular disease (aortic and mitral prosthetic valve failure) | 1 | 0 (0.0%) |  |
|  | — Degenerative valvular disease (mitral and tricuspid regurgitation) | 1 | 0 (0.0%) |  |
|  | — Degenerative valvular disease (mitral prosthetic valve failure) | 1 | 0 (0.0%) |  |
|  | — Degenerative valvular disease (tricuspid regurgitation) | 1 | 0 (0.0%) |  |
| Thrombus evaluation |  | 3 | 3 (100.0%) |  |
|  | — LA thrombus | 1 | 1 (100.0%) | Surgical thrombectomy added (n = 1) |
|  | — Pulmonary embolization | 1 | 1 (100.0%) | Surgical thrombectomy added (n = 1) |
|  | — TEE-negative for thrombus | 1 | 1 (100.0%) | Surgical thrombectomy omitted (n = 1) |

Supplemental Table 1. Preoperative-stable cohort: management impact of POCUS-TEE by indication with nested TEE-derived diagnoses.

Rows are grouped by clinical indications (top level) with corresponding diagnoses nested underneath. Values are n (%) for Surgical plan changed after POCUS-TEE; percentages use the row total as the denominator. The rightmost column lists the surgical plan change observed within each row (ties allowed).

Abbreviations: POCUS-TEE, point-of-care transesophageal echocardiography; RV, right ventricle; LA, left atrium.

| **Indication** | **Diagnosis** | **Total Number** | **No change, n (%)** | **Non-surgical change, n (%)** | **Surgical/Procedural, n (%)** | **Change types** |
| --- | --- | --- | --- | --- | --- | --- |
| MCS evaluation |  | 40 | 8 (20.0%) | 24 (60.0%) | 8 (20.0%) |  |
|  | — Inappropriate pump speed setting | 24 | 0 (0.0%) | 24 (100.0%) | 0 (0.0%) | MCS pump speed adjustment (n = 24) |
|  | — MCS weaning feasible | 8 | 0 (0.0%) | 0 (0.0%) | 8 (100.0%) | MCS weaning (n = 8) |
|  | — TEE-negative for cannula malposition | 4 | 4 (100.0%) | 0 (0.0%) | 0 (0.0%) |  |
|  | — MCS weaning not feasible | 3 | 3 (100.0%) | 0 (0.0%) | 0 (0.0%) |  |
|  | — RVAD-associated pulmonary valve regurgitation | 1 | 1 (100.0%) | 0 (0.0%) | 0 (0.0%) |  |
| Thrombus evaluation |  | 19 | 10 (52.6%) | 5 (26.3%) | 4 (21.1%) |  |
|  | — No thrombus detected | 10 | 10 (100.0%) | 0 (0.0%) | 0 (0.0%) |  |
|  | — Left atrium thrombus | 8 | 0 (0.0%) | 5 (62.5%) | 3 (37.5%) | Anticoagulation initiation/intensification (n = 5); Surgical thrombectomy (n = 3) |
|  | — Left ventricle thrombus | 1 | 0 (0.0%) | 0 (0.0%) | 1 (100.0%) | Surgical thrombectomy (n = 1) |
| Ischemia evaluation |  | 14 | 11 (78.6%) | 1 (7.1%) | 2 (14.3%) |  |
|  | — No new regional wall-motion abnormality | 9 | 9 (100.0%) | 0 (0.0%) | 0 (0.0%) |  |
|  | — Acute myocardial infarction | 2 | 0 (0.0%) | 0 (0.0%) | 2 (100.0%) | CABG (n = 2) |
|  | — Aortic stenosis | 1 | 1 (100.0%) | 0 (0.0%) | 0 (0.0%) |  |
|  | — Left ventricular failure | 1 | 0 (0.0%) | 1 (100.0%) | 0 (0.0%) | Inotropic therapy (n = 1) |
|  | — Aortic regurgitation | 1 | 1 (100.0%) | 0 (0.0%) | 0 (0.0%) |  |
| Pericardial effusion evaluation |  | 9 | 9 (100.0%) | 0 (0.0%) | 0 (0.0%) |  |
|  | — Pericardial effusion without hemodynamic compromise | 9 | 9 (100.0%) | 0 (0.0%) | 0 (0.0%) |  |
| Shunt evaluation |  | 6 | 4 (66.7%) | 1 (16.7%) | 1 (16.7%) |  |
|  | — Residual ventricular septal perforation | 4 | 3 (75.0%) | 0 (0.0%) | 1 (25.0%) | Re-VSP repair (n = 1) |
|  | — Patent foramen ovale | 1 | 0 (0.0%) | 1 (100.0%) | 0 (0.0%) | Ventilator adjustment (n = 1) |
|  | — Ventricular septal defect | 1 | 1 (100.0%) | 0 (0.0%) | 0 (0.0%) |  |
| Aortic dissection evaluation |  | 5 | 5 (100.0%) | 0 (0.0%) | 0 (0.0%) |  |
|  | — TEE-negative for aortic dissection | 4 | 4 (100.0%) | 0 (0.0%) | 0 (0.0%) |  |
|  | — Known type B aortic dissection, patent false lumen | 1 | 1 (100.0%) | 0 (0.0%) | 0 (0.0%) |  |
| Valve evaluation |  | 5 | 4 (80.0%) | 0 (0.0%) | 1 (20.0%) |  |
|  | — Normal prosthetic valve function | 2 | 2 (100.0%) | 0 (0.0%) | 0 (0.0%) |  |
|  | — Recurrent aortic valve regurgitation after aortic valve repair | 1 | 0 (0.0%) | 0 (0.0%) | 1 (100.0%) | Valve replacement (n = 1) |
|  | — TEE negative for infective endocarditis recurrence | 1 | 1 (100.0%) | 0 (0.0%) | 0 (0.0%) |  |
|  | — Systolic anterior motion after mitral valve repair | 1 | 1 (100.0%) | 0 (0.0%) | 0 (0.0%) |  |
| TEE-guided pulmonary artery catheter placement |  | 2 | 2 (100.0%) | 0 (0.0%) | 0 (0.0%) |  |
|  | — PA catheter insertion assist | 2 | 2 (100.0%) | 0 (0.0%) | 0 (0.0%) |  |
| Coronary sinus hematoma evaluation |  | 1 | 1 (100.0%) | 0 (0.0%) | 0 (0.0%) |  |
|  | — Coronary sinus hematoma | 1 | 1 (100.0%) | 0 (0.0%) | 0 (0.0%) |  |

Supplemental Table 2. Postoperative-stable cohort: management impact of POCUS-TEE by indication with nested TEE-derived diagnoses.

Rows are grouped by clinical indications (top level) with corresponding diagnoses nested underneath. Values are n (%) for No change, Non-surgical change, and Surgical/Procedural intervention after POCUS-TEE; percentages use the row total as the denominator. The rightmost column lists the management change categories observed within each row (ties allowed). Abbreviations: POCUS-TEE, point-of-care transesophageal echocardiography; MCS, mechanical circulatory support; RVAD, right ventricular assist device; PA cath, pulmonary artery catheter; CABG, coronary artery bypass graft; VSP, ventricular septal perforation.


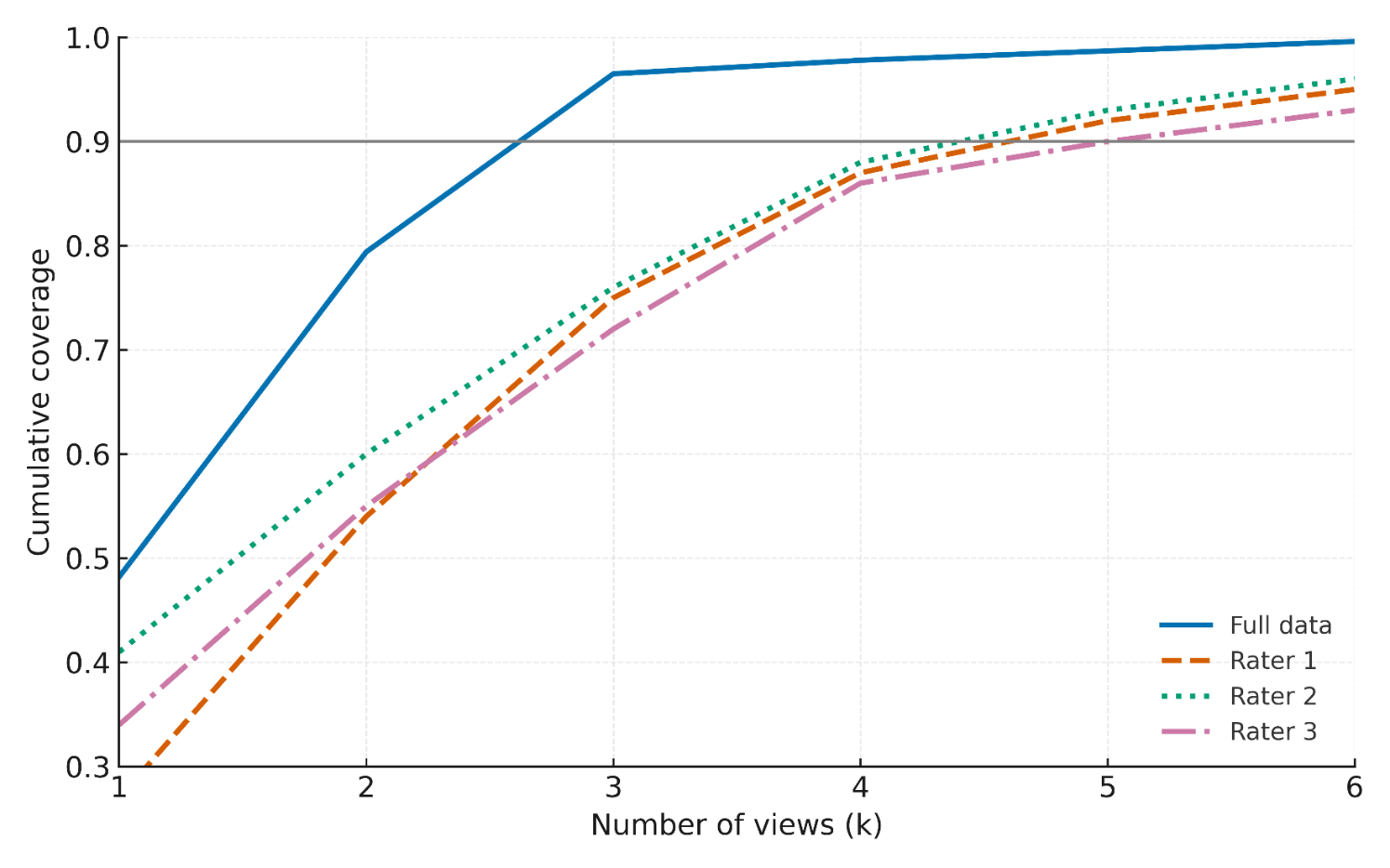


Supplemental Fig. 1. Cumulative coverage as a function of the number of views (k). Lines depict cumulative coverage, the proportion of examinations whose first informative view is contained within the top-k most frequent views, for the full cohort (solid blue) and for three independent raters (orange dashed, green dotted, and purple dash-dot). Abbreviations: k, number of views included.
